# Neonatal and Pediatric Transport: A Contemporary Review

**DOI:** 10.3390/children13020175

**Published:** 2026-01-27

**Authors:** Keith Meyer, Balagangadhar R. Totapally

**Affiliations:** 1 Division of Critical Care Medicine, Nicklaus Children’s Hospital, Miami, FL 33155, USA; balagangadhar.totapally@nicklaushealth.org; 2 Herbert Wertheim College of Medicine, Florida International University, Miami, FL 33155, USA

**Keywords:** neonatal transport, pediatric transport, critical care transport, quality and safety, ECMO transport, telemedicine, transport physiology, pediatric critical care

## Abstract

Safe transport of critically ill infants and children is an essential component of high-quality, pediatric regionalized care. The modern transport environment blends principles of critical care medicine, aviation physiology, provider training, and coordinated systems of care. This review provides an updated examination of current practices in neonatal and pediatric transport, including team structure, clinical bundles, operational considerations, and emerging technologies. Special attention is given to rapidly evolving areas, including data-informed dispatch, real-time clinical decision support, and next-generation devices. The review closes with a discussion of future priorities for research, workforce development, and system design.

## 1. Introduction

Neonatal and pediatric transport is a distinct, highly specialized field within critical care medicine. It requires immediate recognition of clinical deterioration, early stabilization, and the ability to deliver continuous intensive care during transfers between facilities [1,2]. Infants and children who require advanced therapies frequently originate in centers that lack the personnel or technology needed for definitive treatment. The transport process, therefore, becomes a critical extension and often lifesaving piece of the receiving intensive care unit. The principles of safe transfer include: a well-trained, experienced team; appropriate equipment and mode of transportation; full assessment and stabilization of the patient before mobilization; monitoring, reassessment, and stabilization during transport; structured handoff; and proper documentation [3].

Over the past two decades, pediatric health systems in the United States have built structured networks that rely on dedicated transport teams [1,2]. These “spoke and hub” networks allow high-risk infants and children to reach specialized surgical, cardiac, trauma, or medical units in a timely manner [4]. This evolution has significantly improved access to high-quality pediatric healthcare across the United States and its surrounding territories, and specialized pediatric transport systems have improved outcomes [5]. The field now reflects a mature synthesis of clinical science, operations management, aviation safety, and family-centered care [6,7,8]. This article is a contemporary review of the state of pediatric/neonatal transport. We have conducted a focused literature search for each section in the article to present relevant information.

## 2. Historical Perspective

Early neonatal and pediatric transports in the 1980s were performed with limited structure, variable staffing models, and limited resources. Teams often worked with improvised equipment, sparse monitoring, and widely variable training. Infants were especially vulnerable to temperature loss, hypoglycemia, and airway complications, all of which occurred with concerning frequency [9]. As neonatal incubators with controlled temperature became available and rotor-wing transport programs expanded, expectations for safe interfacility transfer began to shift. The parallel development of dedicated pediatric intensive care units also highlighted the need for more organized and clinically capable retrieval teams [7].

The earliest documented transport of a preterm infant by helicopter occurred in 1967 [10]. By the 1990s and early 2000s, a more formal transport model had emerged [1]. Hospitals increasingly recognized that outcomes improved when infants and children were transported by clinicians who understood neonatal and pediatric physiology and were specifically trained for this environment [5]. Published studies reinforced this point, showing reductions in preventable complications and improved overall outcomes when specialized teams were utilized [2,5]. Accreditation organizations, most notably the Commission on Accreditation of Medical Transport Systems (CAMTS), helped define standards for training, clinical competencies, risk management, and communication practices [6,11]. The second National Pediatric and Neonatal Interfacility Transport Medicine Leadership Conference in 2000 addressed issues related to team configuration, the medical director role, accreditation, and benchmarking, which led to recognition of transport medicine as a developing specialty [7]. This period marked the transition from an improvised system to a consistent, professionalized field.

Moving to the past decade, capability has expanded in an almost logarithmic fashion. Modern programs are now capable of routinely transporting infants and children on noninvasive and invasive ventilation, high-frequency oscillatory ventilation (HFOV), portable nitric oxide, therapeutic hypothermia devices, advanced monitoring systems, and even extracorporeal life support [12,13,14,15]. Point-of-care ultrasound has become a practical tool for airway confirmation, vascular access, and hemodynamic assessment [16]. Notable advances in monitoring, including continuous CO_2_ monitoring, have occurred over the last two decades [12,17]. The rise in national and international registries, along with structured quality collaboratives, such as the Ground and Air Medical Quality in Transport (GAMUT) quality improvement collaborative, has brought greater clarity to trends in outcomes, safety priorities, and best practices [1,2,7,13,18]. These developments have positioned neonatal and pediatric transport as a mature and essential component of contemporary neonatal and pediatric critical care medicine.

## 3. Rationale for Specialized Transport Teams

The clinical rationale for dedicated teams is straightforward. Infants and children are vulnerable to rapid physiologic deterioration. Low body mass, immature autonomic regulation, and narrow margins for error contribute to risk. A well-prepared, specialized team with high-quality training, skills, process, and medical leadership significantly improves outcomes. It reduces secondary injury from events like hypothermia, hypoglycemia, hypoxia, hypotension, and delayed recognition of clinical decline [5]. Guidelines from the American Academy of Pediatrics (AAP) and the American College of Surgeons emphasize that thoughtful design, pre-planning, and monitoring of the transport process improve outcomes for critically ill children [11,19].

Specialized teams also bring system-level advantages. They ensure consistent communication with referring providers, predictable handoff processes at receiving centers, and standardized checklists that reduce preventable variation [2,19,20]. Programs with robust data systems demonstrate improvements in temperature control, airway stability, medication accuracy, and patient experience.

More than 17 million children live at least an hour from a regional children’s hospital. Transport teams play an important role in promoting equitable care [11,20,21]. In many regions, they serve as the only reliable link between rural hospitals and advanced neonatal or pediatric centers. When supported by telemedicine, they can guide early stabilization even before dispatch [22,23].

## 4. Transport Activation, Referral, and Triage

Efficient activation begins with clear referral pathways, interfacility transfer agreements, and explicit consultation thresholds [20]. Decision support tools, within a system-based approach, help referring providers assess severity, required capabilities, and the appropriateness of the destination [24]. Triage tools are helpful in selecting appropriate patients, determining the transport team composition and mode, and selecting the destination. The Pediatric Transport Triage Tool was developed to objectively guide selection of team composition and transport mode, thereby standardizing transport planning [25]. Another triage tool, the Queensland Paediatric Transport Triage Tool, demonstrated high accuracy in predicting the need for retrieval or intensive care admission, with a sensitivity of 96.9% for predicting transport by a retrieval team and 96.8% for predicting intensive care admission [26].

Transport stabilization guidelines and bundles that address temperature, glucose, vascular access, and initial respiratory support improve patient readiness for transfer. The AAP encourages transport program leadership to develop and implement policies and procedures for patient care during transport, including annual transport education incorporating equipment training, didactic education, simulation, and skills verification of low-volume, high-risk procedures consistent with the types of care provided during transport [19]. Standardized consultation notes and order templates prevent omissions.

Destination planning requires coordination with bed management systems and subspecialty teams. Centers with cardiac, surgical, trauma, or extracorporeal support programs often rely on real-time triage conferences that include intensivists and transport leadership.

In the United States, compliance with the Emergency Medical Treatment and Labor Act (EMTALA) governs the administrative framework for interfacility transfer [27]. EMTALA requires Medicare-participating hospitals (including critical access hospitals) to arrange for transport to a higher level of care if appropriate emergent care cannot be provided [28]. Documentation of sending and accepting teams, informed consent, and medical necessity is essential.

## 5. Team Composition and Competency

Transport team structures differ across programs but share common elements that assure high quality and safety [29]. Most teams are built around providers with focused neonatal or pediatric expertise. Experienced neonatal and pediatric nurses frequently serve as the primary clinicians, with a second nurse, transport respiratory therapist to ensure adequate support for airway management and ventilatory care [29]. In larger systems and high-volume regions, Emergency Medical Technicians, paramedics, or flight paramedics contribute important skills in navigation of the transport environment, patient handling, and operational safety [29]. There is a wide variation in the team composition of neonatal transport teams in a national survey [18]. Less than 15% of transport teams include physicians as standard team members [29]. Teams with at least two clinicians providing care consistent with critical care in a tertiary facility are associated with lower rates of adverse events [17]. The AAP and the Society of Critical Care Medicine (SCCM) guidelines recommend that the transport teams have expertise in providing the emergency and critical care services required by the patient during transport [30].

Some centers incorporate nurse practitioners or physicians for the most complex transports [29]. These teams are often activated for patients with unstable physiology, anticipated need for advanced procedures, or dependency on technologies such as inhaled nitric oxide, ventricular assist devices, or extracorporeal support. In select situations, this higher-level staffing allows immediate intervention during unexpected deterioration and smoother coordination with surgical or ECMO teams on arrival [31]. Team composition has been shown to influence performance scores during simulation [32]. However, a retrospective study found no difference in adverse effects among different pediatric transport compositions [33]. With appropriate training, critical care nurses and paramedics achieved high levels of successful intubation during transportation [34].

Competency expectations within modern transport programs extend well beyond basic life support [11,19]. Core skills include advanced airway management, invasive and noninvasive ventilatory support, vascular access, medication preparation and infusion management, hemodynamic assessment, and thermal control. Transport clinicians must also be proficient with equipment that behaves differently in the mobile environment, including ventilators affected by altitude and vibration, incubators with servo control, and multi-parameter monitors designed for limited space and power. To maintain these skills, many programs require frequent, high-fidelity competency-based training in neonatal resuscitation, pediatric life support, transport critical care, and ultrasound-guided procedures [6,35].

Sustained readiness depends on regular practice. High-fidelity simulation, structured debriefing after missions, and training in human factors enable teams to refine performance under real-world conditions [19,29]. In situ simulation training has been shown to improve provider confidence, enhance training fidelity, and may help onboard new transport team members [36]. Simulation-based training improves team members’ knowledge [37]. Programs that cultivate psychological safety, encourage open communication, and address fatigue risk and workload challenges consistently report stronger team cohesion and fewer operational errors [2]. As patient acuity rises, these elements have become essential components of a reliable and resilient transport system.

## 6. Modes of Transport

Ground, rotor-wing (helicopter), and fixed-wing aircraft are the primary modalities for neonatal and pediatric transport, with each offering distinct advantages based on distance, urgency, and patient acuity (Table 1) [18,38,39,40,41]. The choice of transport depends on several factors, including the nature of the illness, urgency, availability, and geographical and climatic conditions, as well as cost [42]. In children with trauma, transporting with a helicopter has been shown to have a survival benefit in severely injured children [43] and not in less severely ill children [44]. In children with sepsis in a single geographic location, outcomes were similar regardless of mode of transportation (ground vs. helicopter) [45]. The distance to the receiving facility is one of the factors, among others, for deciding the mode of transport [46]. Ventilation management was similar across all three modes of transportation for ventilated children with neurologic conditions [47]. Ground transport remains the most common modality due to broad availability and predictable access. Modern ground units use stretcher systems with integrated power, oxygen, and mounting points that can safely support incubators and ventilators [6].

Rotor-wing transport provides rapid access for short- and intermediate-distance flights, though operations are constrained by weather, visibility, and landing infrastructure. Rotor-wing crews must be mindful and carefully manage altitude-related effects on oxygenation and gas expansion [9].

Fixed-wing aircraft offer greater range and international transport and provide the most controlled environment. Pressurization strategies may be adjusted for infants with pulmonary disorders or for postsurgical cardiac patients who are sensitive to changes in intrathoracic pressure. Teams participating in international transports must always be vigilant to local capabilities, power sources, medication preparations, pumps, as well as laws, rules, and regulations [9].

Marine and specialty transport modes have been used by the US Coast Guard and similar government/ military organizations in remote settings and during disaster response. The Critical Care Air Transport Team (CCATT) was developed by the United States Air Force (USAF) to provide wartime critical care transport of casualties, and it transported 934 Operation Iraqi Freedom and 3605 Operation Enduring Freedom critically ill or injured patients between 2007 and 2018 [48,49]. There is limited published literature on the role of government agencies, such as the Coast Guard and the military, in international civilian transportation.

## 7. Transport Physiology and Environmental Stressors

**Figure 1:**
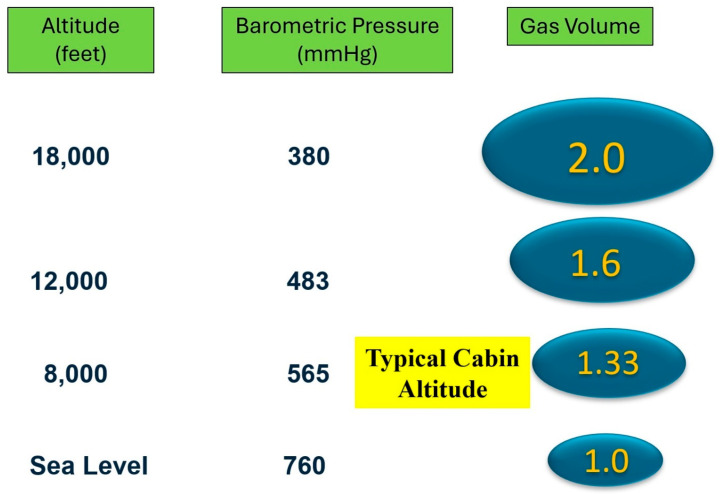
Proportion of gas volumes in cavities at various altitudes and barometric pressures compared to sea level barometric pressure.

Transport exposes patients to unique physiologic challenges [50]. Cabin altitude affects inspired oxygen concentration with increasing altitude and contributes to the expansion of gas in the chest, bowel, or endotracheal tube cuffs [6,9,51]. At cabin pressures of 5000 to 8000 feet, a lower partial pressure of oxygen can lead to decreased oxygen tension in the cerebral and spinal circulations [52,53]. There are several risk factors for flying at higher altitudes, including trapped air in various organs and cavities [54]. Flying at cabin altitude may increase gas volume by 33% according to Boyle’s Law (Figure 1). The cuff size of the endotracheal tube may increase during high-altitude flight, with implications for prolonged transportation [55,56,57]. It is advisable to fly at a cabin pressure below 500–1000 feet for any patients with trapped gases [58]. The transport medical director may also need to advise the team to fly at lower cabin altitudes when transporting children and neonates with certain risk factors [54]. Flying at lower cabin altitude is associated with other issues, such as increased turbulence, higher fuel consumption, and reduced aircraft range.

Motion, vibration, acceleration, and deceleration forces, as well as limited space availability, influence hemodynamic stability, monitoring accuracy, medication delivery, and other interventions [2,11,59,60].

Neonates transported by both rotary wing air and ground ambulance are exposed to sound levels exceeding published recommendations (up to 86 dBA in air transport) and vibration levels above adult standards [50,59,60,61]. Noise levels create communication barriers, which must be addressed through standardized verbal checks and closed-loop responses.

Thermoregulation is critical, especially in neonates. Newborns lose heat rapidly through radiation, convection, and evaporation. Servo-controlled (controlled with a feedback loop) incubators with humidity control reduce these risks, while premature infants require additional measures to maintain temperature stability [62,63,64]. Newborns, especially preterm and low birth weight infants, are highly susceptible to hypothermia and hyperthermia during transport. Hypothermia rates remain significant, particularly in infants < 1000 g, and are associated with increased morbidity and mortality [62,65,66,67].

Infectious risks have become more prominent since the COVID era. Programs increasingly rely on stepwise personal protective equipment protocols, supervised doffing procedures, and specialized containment devices for high-risk pathogens [6].

## 8. Equipment and Clinical Capability

Modern transport platforms include high-tech integrated power systems, redundant oxygen supplies, multi-parameter monitors, and devices that mirror the capabilities of an intensive care unit [17,68,69]. Ventilators used during transport must tolerate altitude changes, vibration, and limited space for adjustments [68,70,71]. Modern transport ventilators can deliver high minute ventilation, high flow, and positive end-expiratory pressure up to at least 20 cmH_2_O [71]. Many can now support conventional ventilation, pressure-control modes, and continuous waveform capnography.

Redundant oxygen supplies with adequate reserves are standard of care, with projected needs plus a 30 min reserve for intrahospital transport [69]. The calculation of oxygen requirements depends on oxygen utilization per minute and the anticipated transport duration (Table 2) [69,72,73,74]. Oxygen concentration must be precisely regulated, particularly for neonates and patients with ductal-dependent congenital heart disease [69]. Underestimation of the oxygen supply needed during transportation can occur, leading to serious problems [75]. Oxygen requirements increase in critically ill children with an increase in altitude because the effective oxygen concentration decreases from 20.9% at sea level to approximately 19.4% at typical helicopter operating altitudes (~2000 ft) and to about 15.4% at typical fixed-wing cabin altitudes (~8000 ft) [71].

Nitric oxide delivery systems have become more compact and reliable, allowing safe transport of infants with persistent pulmonary hypertension. Transport teams have successfully implemented iNO delivery during both ground and air transport, with methods varying by transport modality [13,72,76]. Use of nitric oxide during transportation has increased [13].

Transporting neonates in resource-poor countries is challenging [77]. Incubators may not be available, and kangaroo-care may be needed for temperature maintenance [78]. Neonatal transport systems remain severely underdeveloped across low-and middle-income countries. Overall mortality among referred neonates reaches 30.1% in some settings, with 52% of deaths occurring within 24 h of arrival [79]. Studies from Tanzania demonstrate that while ambulances are used in approximately 88.5% of neonatal transfers, critical deficiencies persist, no ambulances have incubators, and only 2.0% of neonates were maintained in the kangaroo mother care position during transport [78]. Similarly, in rural Tanzania, only 1.9% of public ambulances had equipment for respiratory and cardiovascular support [77]. These inadequacies contribute to poor clinical status on arrival, with 33% of transferred neonates arriving hypothermic, 21.3% hypoxic, and 14.1% hypoglycemic [78]. Five key challenges identified in the referral system include inadequate equipment, low skill level of the transfer team, lack of a centralized transport unit, unclear information exchange, and transport models constrained by administrative structures [80]. Systematic reviews emphasize that safe neonatal transfer, alongside training and effective implementation of proven interventions, is urgently needed to reduce mortality [81].

Point-of-care ultrasound (POCUS) enables teams to confirm line position, assess cardiac function, and troubleshoot endotracheal tube placement [82,83]. POCUS is superior to radiography for assessing the position of the umbilical venous catheter tip and catheter migration, enabling real-time visualization and adjustment. Current evidence strongly supports POCUS-guided internal jugular venous catheter placement and arterial line placement, with demonstrated improvements in first-attempt success rates and reduced complications [84]. Most literature to date has examined adult use in pre-hospital and critical care environments; however, recent studies have begun to demonstrate expanding support for POCUS in pediatric and neonatal transport [85].

Medication management depends on standardized drug kits, color-coded organization, and meticulous infusion pump checks [68]. The list of essential medications is published by SCCM transport guidelines [69]. Specialized medications may be required when transporting patients with specific needs. Redundancy is essential in preparing for successful transport [17,68]. Spares for oxygen, battery power, and critical medications reduce the risk of failure.

## 9. Clinical Bundles and Condition-Specific Care

Neonatal transport guidelines and bundles emphasize temperature control, glucose management, and sepsis pathways that encourage early antibiotics when indicated [6,86,87,88].

Therapeutic hypothermia with servo-controlled devices and meticulous temperature monitoring should be offered during transport for newborns ≥ 36 weeks’ gestational age with moderate-to-severe hypoxic–ischemic encephalopathy [6,89,90].

Transport of children with respiratory failure can now be safely supported with noninvasive and invasive respiratory modalities. Management of bronchiolitis, status asthmaticus, and pediatric acute respiratory distress syndrome must consider both transport physiology and expected disease trajectory [9].

Neonatal cardiac patients are likely the most fragile, especially those with duct-dependent lesions, often requiring prostaglandin infusion, careful oxygen titration, and attention to systemic perfusion [6]. Elective postnatal transfer of neonates prenatally diagnosed with duct-dependent lesions appears to be safe [91]. The decision to intubate neonates receiving low-dose prostaglandin E2 infusion before transport is a clinical one, based on the neonate’s condition, transport time, and team capabilities. In certain neonates, it appears safe to transport them without intubation [92,93]. In low- and middle-income countries, neonates with congenital heart disease often reach referral centers in suboptimal condition following transport, which is associated with adverse clinical outcomes [94]. Integrated robust transport systems may improve outcomes for these patients [94].

Neurocritical patients benefit from controlled ventilation, seizure management, and avoidance of hypotension and hypoxia. Delayed treatment of status epilepticus is associated with adverse outcomes. A multicenter study showed that pre-hospital treatment of children presenting with refractory status epilepticus by the Emergency Medical Services could be streamlined [95]. Transport teams should be equipped to monitor and manage neurocritical care patients during intra- and inter-hospital transfers [60].

Children with shock or sepsis require early vasoactive support and careful fluid management [96,97,98]. Trauma patients require thoughtful immobilization that respects pediatric anatomy and physiology [99,100]. If emergency intubation is needed, a cervical collar may make safe intubation difficult, and in-line manual immobilization may be preferred [101]. Meticulous ventilation management and monitoring to avoid hypoventilation as well as hyperventilation are important for transporting neurocritical care patients [47].

## 10. Pre-Transport Stabilization

There are two main philosophies in pre-mobilization stabilization by the transportation teams: “scoop-and-run” vs. “Stay-and-play”. Each has its own pros and cons. The approach depends on the transport distance, the skill level of the transport teams, the location of retrieval (field vs. hospital), and the urgency of the need for definitive care [102]. R. Adam Cowely is credited with advocating the “golden hour” rule for trauma retrieval in the 1970s [103]. The scoop-and-run philosophy in transport emanates from the concept that delaying definitive care is harmful. In urban centers with short transport distances, it is advised to perform minimal interventions in the field and to transport trauma patients to the nearest trauma center for definitive care. Whereas in rural conditions and where transport time is longer, more advanced interventions and stabilization may be needed [104]. A prolonged period of field stabilization of severely injured patients before reaching the hospital for definitive treatment may be harmful [105].

The scoop-and-run practice may not be appropriate for transporting neonates and children from another hospital by a specialized transport team with a higher level of skill [106]. For pediatric interfacility transports, current evidence may not support a scoop-and-run strategy [69]. The American College of Critical Care Medicine emphasizes that referring hospitals should perform necessary evaluation and stabilization prior to transfer to optimize safety during transport [69]. In one study, pre-hospital interventions by the emergency medical service, when appropriate, did not delay transport or outcomes [107]. With advances in technology and team training, the golden hour rule needs to be redefined [108]. Early goal-directed stabilization before transport to a tertiary care children’s hospital, along with bringing the expertise to the child, will improve outcomes [108].

Sometimes, optimal stabilization is not feasible, and urgent definitive treatment is needed, which is offered at the receiving hospital. In such cases, stabilize the child to the best extent possible and transport to the hospital where definitive care can be provided. Some of the conditions where the scoop and run approach may be adopted include surgical emergencies (e.g., abdominal surgical emergency with non-accidental injury), neurosurgical emergencies (e.g., epidural hematoma, hydrocephalus with raised intracranial pressure), cardiac surgical emergencies (transposition of great arteries not responding to prostacyclin infusion), and pre-ECMO conditions. Even in these conditions, basic stabilization, securing the airway, establishing working intravenous access, and continued stabilization during transport should be done.

## 11. Communication, Family Engagement, and Handoffs

**Figure 2:**
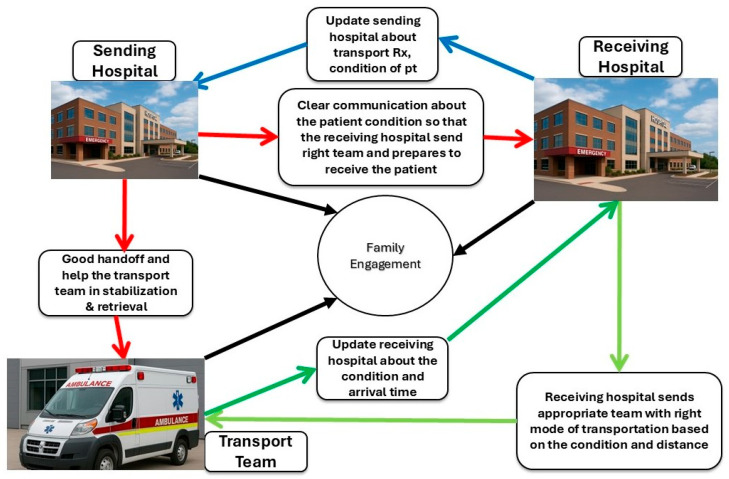
Closed-loop communication between the sending hospital team, receiving hospital team, transport teams, and family.

**Figure 3:**
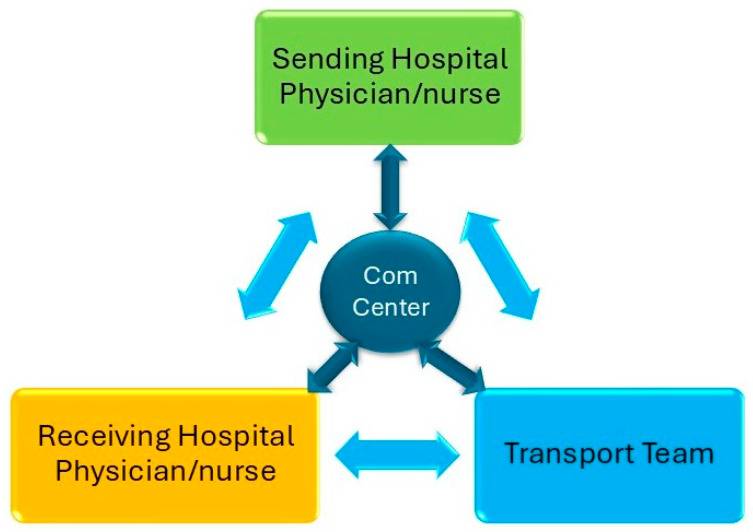
Communication channels among the sending hospital, the receiving hospital, and the transport team. Com center = Communication center.

Effective communication, family engagement, and structured handoffs are critical components of safe neonatal and pediatric transport, supported by multiple American Academy of Pediatrics guidelines and recent evidence demonstrating improved outcomes and parental satisfaction [6]. The specialty has long adopted practices from aviation and other high-reliability fields, where predictable processes, explicit verbal exchanges, and shared situational awareness reduce the risk of error. In transport medicine, the communication chain begins even before the team launches (Figure 2). Communication channels between the sending hospital, receiving hospital, and the transport team can be direct or through a communication center (com center) (Figure 3).

Implementation of a modified SBAR (Situation, Background, Assessment, Recommendation) tool for neonatal transport significantly improved the quality of clinical information shared between multidisciplinary team members, with improvements in both global scores and cumulative scores [109]. Collaborative decision-making between referring and accepting physicians is essential to determine the transport mode, the level of team expertise, and the necessary equipment, while considering both patient safety and family impact [24]. A structured pre-arrival briefing helps ensure that the transport team arrives with a unified plan. These briefings typically include a review of the patient’s condition and trajectory, anticipated interventions, the potential need for airway or vascular access procedures, and confirmation that critical equipment and medications are available and functioning. This approach mirrors the safety culture emphasized in the AAP Guidelines for Air and Ground Transport of Neonatal and Pediatric Patients, which highlight role clarity, preparation, and standardized language as core elements of transport readiness [6].

Communication with the referring facility is equally central. The initial consultation should clearly outline the reason for transfer, the key events leading to deterioration, current therapies and ventilator settings, and any notable challenges such as difficult airway anatomy, challenging intravenous access, risk of instability during handling, or specific family concerns [24]. Interdisciplinary consultations, for example, to understand infection-control precautions for neonatal transport in the event of maternal COVID-19 infections [110,111]. When available, telemedicine can add important detail by allowing the transport physician or team leader to visually assess the patient, equipment, and environment prior to departure. This early exchange often leads to more targeted stabilization and faster control of temperature, glucose, and respiratory status before the team arrives [9].

Parental involvement and communication are fundamental to family-centered transport. Family engagement is a defining feature of pediatric transport. Recent studies reveal that only 53% of parents traveled with their babies during transport, and only 52.6% felt involved in the transport process [112]. Parents consistently identify key areas for improvement, including the possibility of parental accompaniment during transport, telephone contact upon arrival at the destination hospital, and enhanced transmission of medical information both prenatally and during transport [113].

For parents, the arrival of a transport team often marks one of the most emotionally intense moments of their child’s illness. Clear, compassionate communication can significantly lessen fear and confusion. Families benefit from understanding who the team members are, what will occur during preparation and travel, how their child will be monitored, and what to expect at the receiving center. The AAP has long emphasized the importance of family-centered care, and many transport programs integrate parents as partners when the clinical situation and mode of transport allow. Parental accompaniment may be possible in selected ground missions and can help maintain emotional continuity for the child.

The handoff at the receiving center completes the communication process. Unstructured verbal reports risk omissions, especially in crowded or time-pressured environments such as emergency departments or intensive care units. Many programs therefore use a simple, standardized handoff structure that conveys the child’s illness severity, a concise summary of the clinical course, interventions performed en route, current therapies, and any anticipated challenges. Whether using a locally developed tool or adapting concepts from validated pediatric handoff frameworks, the goal remains the same: to deliver a clear, complete, and organized report that allows the receiving team to assume immediate responsibility with full situational awareness. Closed-loop confirmation, in which the receiving team repeats back critical details or asks targeted clarifying questions, reduces the risk of misunderstandings.

These communication practices reinforce the broader safety culture that supports neonatal and pediatric transport. Consistent pre-mission briefings, clear dialogue with referring clinicians, supportive engagement with families, and structured handoffs at the receiving center all contribute to smoother transitions, fewer preventable errors, and better patient experience. As programs integrate newer technologies, including emerging artificial intelligence, real-time data transfer, and standard quality measures such as Ground and Air Medical Quality in Transport (GAMUT) metrics, communication is expected to become even more reliable and more closely aligned with the principles of high-quality pediatric care [19,114,115].

## 12. Safety, Quality, and Risk Management

Critically ill patients can be transported safely with modern equipment, specialized training, and proper planning. However, there is an inherent risk in transportation, and typically, anything that can go wrong can [116]. Medical complications, equipment failures, and technical problems can occur during transport [117]. Early identification of vulnerable pediatric patients could improve patient stability during transport. Higher nursing care complexity was independently associated with an increased likelihood of transfer events and can be used to identify patients for early transfer [118]. Safety is the foundation of neonatal and pediatric transport. Programs operate in dynamic, unpredictable environments, where minor lapses in communication, preparation, or equipment management can cause significant harm. For this reason, modern transport systems place deliberate emphasis on standardized processes, continuous risk assessment, and reliable methods for identifying and correcting system weaknesses. Measuring the performance is important for any transport system [119]. Robust, continuous monitoring of safety and performance should be part of every transport program.

### 12.1. Safety Culture and Human Factors

The AAP Guidelines for Air and Ground Transport of Neonatal and Pediatric Patients highlight the importance of a strong safety culture rooted in clear expectations, role clarity, and open communication [6,9]. Transport teams operate in conditions that require rapid decision-making and coordinated action. Human factors training, including situational awareness, workload management, and error-prone moment recognition, has become central to daily operations. Regular pre-mission briefings, equipment checks, and challenge-response confirmations create predictable behaviors that reduce the chance of oversight [6].

The reported incidence of adverse events during intrahospital transfers varied across studies, which included emergent tracheostomy, pneumothorax, and cardiac arrest requiring chest compressions [120]. Respiratory and airway complications were the most frequently encountered, and hypothermia was particularly common among infants [120]. Most adverse events during intrahospital transport of anesthesia patients are preventable [121]. Countermeasures for several error traps in intrahospital transfer of these patients include multidisciplinary planning and optimal resource utilization, the use of structured tools, standardized communication, and checklists to improve care processes, fostering a strong culture of safety during transport, and adjusting team composition and leadership style to match the specific clinical situation [122].

“Just Culture” principles encourage team members to report near misses, equipment concerns, or communication failures without fear of blame [122,123,124,125]. Programs that actively promote psychological safety are more likely to identify emerging risks early and prevent them from reaching patients.

### 12.2. Quality Improvement and Performance Metrics

Quality improvement efforts have strengthened considerably over the past decade. Many transport programs now participate in structured databases and benchmarking collaboratives, most notably the GAMUT initiative. The GAMUT provides a set of transport-specific quality indicators with established definitions, allowing programs to measure performance in a meaningful and comparable way [126].

Metrics commonly used in neonatal and pediatric transport include

- First-attempt endotracheal tube success without hypoxia or hypotension.
- Use of continuous waveform capnography for advanced airways.
- Time from acceptance to team mobilization.
- Avoidance of unplanned device dislodgement.
- Temperature and glucose stability on arrival.
- Safe administration of vasoactive infusions and other high-risk medications.

These indicators focus on clinical care, operational reliability, and safety events. Because GAMUT stratifies metrics by age group, programs can evaluate neonatal and pediatric performance separately and track improvements over time. The integration of GAMUT has provided transport medicine with one of the most practical and meaningful quality structures in pediatric critical care [9,35,115].

### 12.3. Operational Safety and Equipment Reliability

Transport missions require multiple layers of redundancy to account for equipment failure, environmental stressors, and unforeseen delays. Routine use of standardized equipment checks, load plans, and documentation templates reduces the risk of missing essential supplies. Programs commonly use device-specific checklists for ventilators, incubators, monitors, medication pumps, and oxygen systems [127]. Equipment must also be routinely evaluated for durability under vibration, temperature variation, and altitude changes.

Many programs maintain close collaboration with fleet operations, aviation partners, and biomedical engineering teams to ensure that stretcher mounts, power systems, and oxygen delivery devices meet safety requirements. These partnerships allow early identification of potential risks such as power instability, depleted oxygen reserves, or design flaws that may affect patient safety.

### 12.4. Incident Review and Feedback Systems

High-performing programs conduct structured reviews of all safety events and selected high-acuity missions. Reviews often include both clinical and operational perspectives and may involve transport clinicians, medical directors, aviation personnel, and referring or receiving providers. Debriefings immediately after mission completion provide timely insight into performance, while more formal case reviews allow for deeper analysis of trends.

Many programs integrate simulation to address identified vulnerabilities, allowing teams to test new processes, refine teamwork behavior, and improve readiness for rare but high-risk situations. Training using simulation has been shown to benefit several aspects of the transport system, including skills training, communication, debriefing techniques, and checklist use, among others [32,128,129,130,131,132,133,134].

### 12.5. Integration of Technology into Safety Systems

Emerging tools are beginning to support safety and quality efforts in transport medicine. These include remote physiologic monitoring [135,136], real-time data streaming from monitors and ventilators [137], integrated documentation platforms, and early warning indicators that identify rising risk during transport. Telemedicine support from intensivists or subspecialists can assist frontline teams during challenging situations, especially when a child deteriorates unexpectedly [21,23,136,138].

As these tools and technologies evolve, careful oversight is required to ensure they enhance rather than distract from clinical judgment. The principles of transport safety, clarity, redundancy, thoughtful preparation, and structured communication remain essential regardless of technological advances.

## 13. Legal and Ethical Considerations

Neonatal and pediatric transport operates within a complex legal and regulatory framework that governs patient safety, interfacility transfers, scope of practice, documentation, and the protection of patient information. These elements shape daily operations and must be understood clearly by clinicians, medical directors, and administrative leadership.

### 13.1. Interfacility Transfer Requirements

In the United States, the Emergency Medical Treatment and Labor Act (EMTALA) establishes the foundational expectations for interfacility transfer [69,139,140,141]. Sending hospitals are required to provide a medical screening examination, initiate stabilizing treatment within the limits of their capability, and arrange transfer when a patient requires services unavailable at that facility. The receiving hospital must have the necessary capability and agree to accept the patient.

Transport teams play an important role in meeting these requirements. They must document the reason for transfer, the patient’s condition at the time of departure, therapies provided, and any events during transport.

### 13.2. Licensure, Credentialing, and Scope of Practice

Transport medicine often crosses municipal, county, and state boundaries, which introduces variation in licensure and scope of practice. Programs must ensure that clinicians hold the appropriate nursing, respiratory therapy, paramedic, advanced practice, or physician licenses for their home base geography. Some states maintain explicit regulations governing transportation and the qualifications required for outside agencies. This has been an area of controversy. Specific questions should be referred to your program or hospital’s legal counsel.

Medical directors are responsible for defining clinical practice guidelines and pathways that govern the team’s clinical authority. In high-acuity environments, clear limits on the scope of practice help prevent unsafe variation and ensure that clinicians have the skills and training required.

### 13.3. Privacy, Documentation, and Communication Security

Transport teams routinely share protected health information with referring and receiving facilities. Communication must comply with privacy regulations, particularly the Health Insurance Portability and Accountability Act (HIPAA) [142]. Most programs use secure communication channels, dedicated transport documentation systems, or encrypted telemedicine platforms to prevent inappropriate disclosure.

Accurate documentation remains essential. The transport record must reflect the patient’s clinical status at transfer, interventions performed, monitoring data, medications administered, and any events that occurred. These details support continuity of care, meet legal standards, and are critical for quality improvement review.

### 13.4. Medication Management and Controlled Substances

Medication administration during transport carries unique regulatory responsibilities. Controlled substances must be stored in secure, tamper-evident systems, accounted for through chain-of-custody documentation, and disposed of in accordance with state and institutional policy. The list of medications includes resuscitation drugs, sedatives and analgesics, vasoactive agents, respiratory medications, and maintenance fluids. In addition, specific medications may be needed based on the clinical conditions, such as surfactant for premature babies and prostaglandin E1 for transposing babies with duct-dependent lesions.

Because many states have specific rules governing who may administer certain medications or perform specific procedures on minors, transport programs must maintain clear protocols and ongoing competency assessments for staff.

### 13.5. Ethical Considerations in Pediatric and Neonatal Transport

Ethical challenges are not uncommon. Decisions about whether a child is stable enough for transport, whether the risks of transfer outweigh the potential benefit, or when a family’s goals of care differ from the expected medical pathway require careful judgment. The AAP has long emphasized respect for family autonomy, honest communication, and transparency in decision-making.

When prognosis is uncertain or the likelihood of benefit is limited, transport teams should involve medical control and leadership to assist in navigation. Palliative transfers, transport for comfort care closer to home, and end-of-life travel may be appropriate in selected cases, but require thoughtful discussion with families and unified agreement between sending and receiving clinicians.

## 14. International and Long Distance Transport

International and long-distance transport of neonatal and pediatric patients requires careful medical, operational, and administrative coordination. Programs frequently work with customs and immigration authorities, embassy or consular offices, and local healthcare systems to ensure the timely clearance of personnel, equipment, and controlled medications. Early communication with aviation partners is also essential, as aircraft pressurization strategies (selection of appropriate cabin pressure for clinical conditions) may need to be adjusted for infants with congenital heart disease, pulmonary hypertension, postoperative lung injury, or conditions sensitive to changes in intrathoracic pressure.

Cultural and linguistic considerations play an important role in these missions. Families may be navigating unfamiliar healthcare environments, and clear communication about expectations, timelines, and receiving center capabilities helps maintain trust. Collaboration with local physicians or international assistance organizations improves continuity of care and reduces uncertainty for families during complex travel.

Long-range transport introduces additional challenges related to equipment and medication compatibility. Ventilators, monitors, incubators, and medication pumps must be compatible with aircraft power systems and able to function reliably during extended flight times, pressure changes, and vibration. Redundant power sources and oxygen reserves are essential. Medication availability may differ across borders, and controlled substances require precise documentation and secure storage that meet the requirements of both the exporting and receiving countries.

Many international transports are coordinated through global medical assistance companies or insurers, particularly when a child becomes ill while abroad or when families seek specialized care at major referral centers. Effective collaboration between these organizations and pediatric transport teams ensures alignment of logistics, clinical expectations, and safety standards throughout the journey [9].

## 15. Economics, Operations, and Program Design

Neonatal and pediatric transport programs must balance clinical capability with operational and financial sustainability. The primary cost drivers include staffing, continuous training, equipment acquisition and maintenance, vehicle or aircraft operations, and dedicated communication and dispatch infrastructure. Programs supported directly by children’s hospitals or health systems often operate within broader institutional budgets, while others rely on blended models that incorporate reimbursement from insurers and government payors.

Operational design centers on reliability and readiness. Dispatch systems must coordinate incoming requests, assess urgency, and match resources to patient needs while accounting for distance, weather, and bed availability at receiving centers. High-performing programs maintain clear activation pathways, second-team availability when feasible, and predetermined contingency plans for surge periods.

Quality and safety efforts, including participation in benchmarking collaboratives such as GAMUT, guide operational priorities and resource allocation. These data help programs identify gaps, justify investments, and demonstrate value to hospital leadership and referring partners [115].

## 16. Future Directions

The next decade will bring significant changes to neonatal and pediatric transport. Key areas include the following:

### 16.1. Data-Informed Dispatch and Decision Support

Programs are beginning to adopt real-time risk scores that combine physiologic data, sending facility capability, and regional resource availability. These tools may assist in triage, anticipate the need for advanced support, and shorten the time to definitive care. Decision support integrated into communication centers can standardize early stabilization and reduce preventable deterioration before the team arrives [14].

### 16.2. Remote Physiologic Monitoring and Telemedicine

Advances in secure data transmission allow transport physicians and subspecialists to view physiologic tracings, ventilator waveforms, and ultrasound images during transport. This may improve early detection of clinical decline and allow more precise adjustment of ventilation or circulatory support. Telemedicine at the bedside before team arrival has already shown value in improving temperature control and respiratory management.

### 16.3. Artificial Intelligence in Transport Medicine

Artificial intelligence will likely reshape almost every element of transport care. Predictive models can assist in identifying infants at risk of hypothermia or hypoglycemia, guide the timing of departure, or estimate the need for specific equipment. Algorithms that analyze sound and waveform data may support earlier recognition of airway obstruction or ventilator asynchrony. Artificial intelligence-driven documentation tools may reduce cognitive load and allow teams to focus more on direct patient care.

### 16.4. Next Generation Devices and Equipment

Ventilators are becoming lighter and more tolerant of altitude and vibration. Battery technology is improving, extending mission duration and reducing the risk of power loss. Compact systems for nitric oxide, therapeutic hypothermia, and invasive monitoring are becoming more reliable.

Advances in biocontainment design will allow safe transport of children with emerging infectious diseases. New stretcher platforms that reduce vibration and improve ergonomics will lower risk for both patients and team members.

### 16.5. Workforce Sustainability and Training

The field faces increasing challenges related to burnout, recruitment, and retention. Programs are developing expanded roles for advanced practice clinicians, structured teleprecepting, and competency-based training pathways. Large-scale simulation networks may eventually allow regional centers to share scenarios and standardize training across systems.

### 16.6. Climate and Disaster Preparedness

Heat events, wildfires, severe storms, and hurricanes are disrupting transport operations with increasing frequency. Programs will need to invest in environmental monitoring, alternate routing plans, and resilient communication systems. The ability to transport children in challenging environments, including smoke-exposed or disaster-affected regions, will become increasingly relevant.

### 16.7. Research Priorities

Key research needs include standardized outcome measures for transport, multicenter trials evaluating transport bundles, improved data on transport physiology, and comparative evaluation of different team models. Human factors research will be essential as teams incorporate artificial intelligence into high-quality care.

Further research is needed to understand the impact of various transport factors, including team composition and training, activation time, transport mode, and transport duration, on patient outcomes. Current evidence suggests that specialized transport systems are associated with better survival outcomes [5,143]. However, the team composition between junior doctors and advanced nurse practitioners has not shown to have any difference in hospital mortality [144]. The relationship between stabilization time and outcomes remains complex. Prolonged stabilization by specialized teams was associated with increased mortality, though this likely reflects residual confounding from illness severity rather than a causal relationship [144]. When evaluating transport time intervals in California’s 47,794 neonatal transports, the time required for evaluation by the transport team was associated with increased risk of clinical deterioration, whereas modifiable transport intervals (dispatch time, travel time) were not [145]. Distance traveled showed no significant association with PICU mortality, even when patients traveled farther than their nearest PICU [143]. Optimization of pre-hospital care and transport protocols is a priority in transport research, as shown in a study of European EMS systems: physician-led transports performed more pre-hospital lifesaving interventions but had longer transport times and higher PICU admission rates and mortality (9.8% versus 1.8%) [146].

## 17. Conclusions

Neonatal and pediatric transport has evolved into a sophisticated extension of critical care medicine. Dedicated teams with specialized training can provide high-quality care during the most vulnerable period of a child’s illness. Continued progress will depend on thoughtful integration of technology, robust data systems, and sustained investment in workforce development. The field is poised for meaningful advances as transport programs incorporate new tools, analytic approaches, and strategies to maintain safety in an increasingly complex environment.

## Figures and Tables

**Table 1:** Advantages, disadvantages, and key indications of three modes of medical transportation.

Mode of Transport	Advantages	Disadvantages	Key Indications
**Ambulance (Ground)**	- Widely available and cost-effective	- Slower over long distances	- Local or regional transfers (<50–100 miles)
	- Ideal for short distances	- Traffic delays	- Stable or moderately unstable patients
	- Easiest to mobilize quickly	- Limited range (<50–100 miles)	- When no aviation assets are available
	- Can accommodate complex equipment (ventilators, incubators, pumps)	- Crew fatigue on long transports	- Weather prohibits flight
	- Minimal weather constraints		
**Helicopter (Rotor-Wing)**	- Fast point-to-point transport	- Limited cabin space; restricted weight/size	- Time-critical emergencies needing rapid intervention
	- No need for runways—can land near hospitals/accident sites	- Weather visibility dependent	- Difficult terrain or inaccessible locations
	- Ideal for time-sensitive emergencies (trauma, stroke, pediatric/neonatal critical care)	- Noise and vibration affect monitoring	- Short to mid-range interfacility transports
	- Bypasses traffic	- Higher cost	- Patient cannot tolerate long ground transport
		- Short-to-medium range (<150–200 miles)	
**Fixed-Wing Aircraft**	- Best for long-distance (>200 miles) or international transport	- Requires runways and ground transport at both ends	- Long-distance interfacility transport (regional, national, international)
	- Can carry more equipment and staff	- Longer mobilization time	- Patients requiring care not available locally
	- More stable flight environment	- Pressurization issues for certain conditions	- Time-sensitive transfers beyond helicopter range
	- Less affected by weather compared to helicopters	- Higher operational cost	- Neonatal/pediatric care needing a smooth/pressurized environment
	- Faster cruise speeds		

**Table 2:** Example: An intubated child with a flow rate of 10 L/min and an expected transport time of 60 min and 50% reserve capacity. (**a**) Calculate total O_2_ required for the transport. (**b**) Check cylinder capacity and number of cylinders.

(**a**)			
**Step**	**Item**	**Symbol/Formula**	**Example Values**
1	Ventilator O_2_ flow	F_1_	10 L/min
2	Additional O_2_ devices (sum if present)	F_2_ … F_n_	0 L/min (none)
3	**Total O** **_2_** **flow**	F_total = F_1_ + … + F_n_	**10 L/min**
4	Planned transport time	T	60 min
5	Safety factor (reserve: 1.3–2.0 commonly)	S	1.5 (50% extra)
6	**Total O_2_ required**	O_2__req = F_total × T × S	10 × 60 × 1.5 = **900 L**
Formula: Total O_2_ needed (L) = (Sum of all flows in L/min) × (Planned time in min) × Safety factor			
(**b**)			
**Step**	**Item**	**Symbol/Formula**	**Example with E Cylinder at 2000 psi**
1	Cylinder factor	CF	0.28
2	Starting pressure	P_start	2000 psi
3	**Cylinder capacity**	Cap = CF × P_start	0.28 × 2000 = **560 L**
4	**Duration per cylinder**	Dur = Cap ÷ F_total	560 ÷ 10 ≈ 56 **min** per E cylinder
5	**Cylinders needed for trip**	N = O_2__req ÷ Cap	900 ÷ 560 ≈ 1.6 → **2 E cylinders**
Formulas: Cylinder capacity (L) = Cylinder factor × (Pressure in psi) ○ (Typical factors: D = 0.16, E = 0.28, M = 1.56) Duration (min) = Capacity ÷ F_total Cylinders needed = O_2__req ÷ Capacity (round up)			

## Data Availability

Not applicable.
